# Fatal malignant pertussis with hyperleukocytosis in a Chinese infant: A case report and literature review

**DOI:** 10.1097/MD.0000000000010549

**Published:** 2018-04-27

**Authors:** Shu-feng Tian, Hong-mei Wang, Ji-kui Deng

**Affiliations:** Division of Infectious Disease, Shenzhen Children's Hospital, Shenzhen, China.

**Keywords:** exchange transfusion, leukocytosis, pertussis

## Abstract

**Rationale:**

Pertussis has re-emerged on a global scale and is an ongoing public health problem, even in countries with high rates of vaccination. Hyperleukocytosis [white blood cell (WBC) count >100 × 10^9^/L] is a rare complication that strongly predicts mortality in cases of severe pertussis.

**Patient concerns:**

We report a case of severe pertussis in an infant who initially presented with persistent cyanotic cough, tachypnea, and grunting. The infant's condition deteriorated rapidly, and she was transferred to the pediatric intensive care unit (PICU) during her third hour of hospitalization. On the third hospital day, her WBC count had increased to 101.85 × 10^9^/L with a lymphocyte count of 36.76 × 10^9^/L, and her hemoglobin level had fallen to 6.9 g/dL. Bone marrow examination found no evidence of tumor cells. Her initial echocardiogram showed no abnormal findings; however, a subsequent echocardiogram 10 days later revealed pulmonary hypertension.

**Diagnoses:**

The patient was diagnosed with severe pneumonia, which was confirmed to be pertussis based on a persistent cough in the infant's mother and the polymerase chain reaction and culture of the infant's nasopharyngeal secretions being positive for *Bordetella pertussis*.

**Interventions:**

The infant was treated with supportive care, early macrolide antibiotics, and broad-spectrum antibiotics before being transferred to the PICU for further management, including continuous venovenous hemodiafiltration.

**Outcomes:**

Unfortunately, the infant died as a result of pulmonary hypertension and multiorgan failure.

**Lessons:**

Exchange transfusion should be considered in all infants who present with severe pertussis with hyperleukocytosis. This guideline is supported by the findings of a comprehensive literature review, which is included in this article, as well as newly published criteria for exchange transfusion therapy. Finally, we hope that adults in China will be vaccinated against *B. pertussis* in order to prevent the infection of infants within their households.

## 1 Introduction

Pertussis (whooping cough) is a highly contagious, vaccine-preventable, respiratory illness caused by *Bordetella pertussis*. In recent years, pertussis infections have re-emerged worldwide.^[1,2]^ Pertussis has now become the most common vaccine-preventable disease.^[3]^ In 2016, the World Health Organization reported 139,535 cases of pertussis, with a mortality rate of 4%.^[4]^ In our department, we treated 595 infants with pertussis between January 1, 2016, and October 31, 2017; only 1 of these infants died. Here, we report the first case of severe pertussis with hyperleukocytosis in a Chinese infant. The patient was a 42-day-old infant, who eventually died as a result of severe complications. The main objectives of this report are to highlight the importance of treating hyperleukocytosis associated with pertussis and to review the literature regarding the usefulness of exchange transfusion (ET) for patients with severe pertussis with hyperleukocytosis.

## 2 Patient information and clinical findings

A 42-day-old (4.8 kg) girl was admitted to the Shenzhen Children's Hospital because of cough, tachypnea, and grunting. She was born at term via normal vaginal delivery with no remarkable neonatal events and exhibited good health at home until 4 days before admission. On examination, she exhibited a respiratory rate of 64 breaths/min; her oxygen saturation was 88% on room air, which improved to 97% upon receiving 3 L of oxygen via face mask. Her heart rate was 156 beats/min, and she demonstrated poor peripheral perfusion. The infant's body temperature was 37.8°C, and she exhibited a poor mental response. Bilateral wheezing and crackles were detected by lung auscultation. The remainder of her physical examination was normal. The infant was diagnosed with severe pneumonia, and she was treated with intravenous cefoperazone-sulbactam and supplemental oxygen by face mask. Pertussis was suspected because of the infant's persistent cyanotic cough, a persistent cough in the infant's mother, and because the infant was too young to have been vaccinated.

## 3 Diagnostic assessment

Polymerase chain reaction (PCR) and culture of the infant's nasopharyngeal secretions were both positive for *B. pertussis*. A chest radiograph on admission showed right lobe collapse and left lobe consolidation. Routine blood tests on the day of admission revealed a hemoglobin (Hb) level of 8.3 g/dL, white blood cell (WBC) count of 77.19 × 10^9^/L, neutrophil count of 35.63 × 10^9^/L, and platelet count of 793 × 10^9^/L; the lymphocyte count could not be determined. Two days later, her WBC count had increased to 101.85 × 10^9^/L with a lymphocyte count of 36.76 × 10^9^/L, her Hb level had fallen to 6.9 g/dL, and a blood smear revealed hyperleukocytosis, anemia, and thrombocytosis. Bone marrow examination found no evidence of tumor cells. The infant's clotting profile was initially normal. Her sodium level was 126 mmol/L, but all other electrolytes were within normal limits. Tests for common pathogens, including *Mycoplasma pneumoniae*, *Chlamydia trachomatis*, respiratory syncytial virus, influenza, cytomegalovirus, Epstein–Barr virus, adenovirus, hepatitis B virus, as well as sputum bacterial cultures, were negative. An echocardiogram found no abnormalities.

## 4 Therapeutic intervention and outcomes

**Figure 1:**
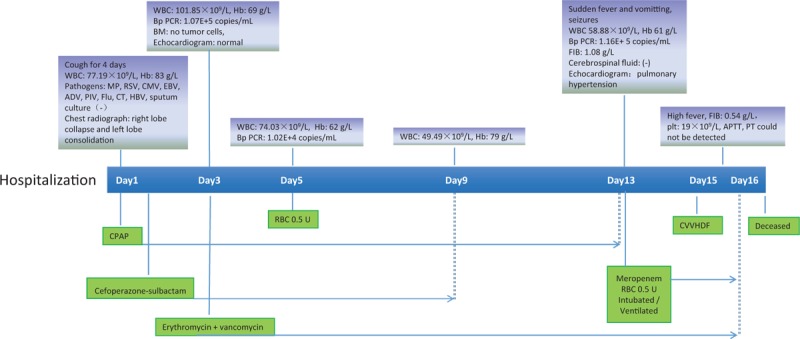
Timeline of important clinical events. ADV = adenovirus, APTT = activated partial thromboplastin time, BM = bone marrow, Bp = Bordetella pertussis, CMB = cytomegalovirus, CPAP = continuous positive airway pressure, CT = *Chlamydia trachomatis*, CVVHDF = continuous venovenous hemodiafiltration, EBV = Epstein–Barr virus, ET = exchange transfusion, FIB = fibrinogen, Flu = influenza, Hb = hemoglobin, HBV = hepatitis B virus, MP = *Mycoplasma pneumoniae*, PCR = polymerase chain reaction, PICU = pediatric intensive care unit, PT = prothrombin time, RBC = red blood cells, RSV = respiratory syncytial virus, WBC = white blood cell.

Due to the patient's worsening respiratory distress in her third hour of hospitalization, she was transferred to the pediatric intensive care unit (PICU) for further management. In the PICU, she was treated with continuous positive airway pressure and underwent 2 transfusions of 0.5 units of packed red blood cells. Intravenous erythromycin and vancomycin were also initiated. On her 13th day of hospitalization, she deteriorated with worsening hypoxemia, generalized tonic seizure, and tachycardia that reached 200 beats/min. She was intubated and ventilated, then treated with phenobarbital and mannitol. Her cerebrospinal fluid and brain computed tomography examinations were normal; her peripheral WBC count remained high, but it gradually dropped to 51.38 × 10^9^/L with 52.5% lymphocytes. Her fibrinogen level dropped to 0.86 g/L, whereas her D-dimer level was significantly elevated at 3060.0 ng/mL. Her liver and kidney function tests were both remarkably abnormal. Her sputum culture was positive for *Streptococcus pneumoniae*. A second echocardiogram revealed severe pulmonary hypertension (pulmonary artery pressure, 60 mm Hg) and an atrial horizontal bidirectional shunt. She underwent continuous venovenous hemodiafiltration (CVVHDF) for 1 day but showed no signs of improvement. Laboratory examinations revealed disseminated intravascular coagulation and multiorgan failure. The infant's parents chose to discontinue treatment on her 16th day of hospitalization, and the patient died soon after being extubated. A timeline of important clinical events is shown in Fig. 1.

## 5 Discussion

An elevated and rapidly rising WBC count is suggested as a predictor of severe *B. pertussis* infection in young infants, making early and repeated WBC count determinations critical in the evaluation of all infants with suspected or proven pertussis.^[5]^ Leukocytosis is associated with the need for mechanical ventilation, pulmonary hypertension, and eventual mortality; pulmonary hypertension may be an independent risk factor of mortality in cases of severe pertussis.^[6]^ An increased WBC count is observed in nearly all cases of pertussis; however, hyperleukocytosis (WBC count >100 × 10^9^/L) is a rare complication of pertussis, which is thought to be caused by the pertussis toxin.^[7]^ This is the first report of pertussis resulting in hyperleukocytosis in a Chinese infant.

Multiple therapies have been described to treat leukocytosis in severely ill infants, including hyperhydration.^[8]^ However, few techniques are available to reduce the leukocyte mass. One such technique, leukapheresis, is a complex technique that requires highly qualified staff, is not available in all hospitals, and may carry a risk of complications.^[9]^ In contrast, ET, which is frequently conducted in the PICU, can be performed by most critical care staff and rarely results in major complications.^[9]^ In 2004, Romano et al^[10]^ published the first report of ET in a patient with severe pertussis; thereafter, ET has been reported in multiple case series and case reports of severe pertussis (Table 1).^[9–22]^

**Table 1:**
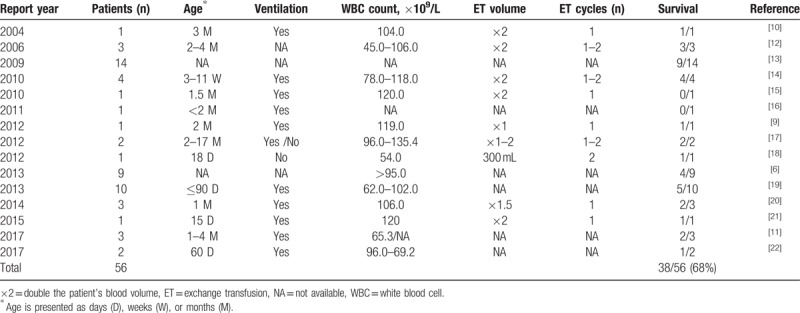
Summary of published pertussis cases in which patients underwent exchange transfusion.

In this case, the patient was suspected to have been infected by a family member. Notably, adults in China are not routinely vaccinated against pertussis, even though most infants (<3 months old) have immediate contact with their parents and other household members. Thus, adult vaccination is extremely important to prevent the transmission of pertussis to infants. In this case, the infant's high WBC count, combined with pulmonary hypertension and multiorgan failure, led us to promptly initiate CVVHDF. Although early macrolide antibiotics and broad-spectrum antibiotics were administered, in conjunction with airway management and other supportive care, unfortunately, the use of CVVHDF did not improve the patient's condition. In cases of extreme leukocytosis, ET may prevent the formation of leukocyte aggregates in the pulmonary vasculature when used early in the course of the disease. ET may also prevent the development of pulmonary hypertension and hemodynamic collapse, thereby improving patient survival rates. Provisional criteria for ET therapy in patients with leukocytosis (lymphocytosis subtype) have been recently suggested.^[11]^ As summarized in Table 1, the survival rate of pertussis patients with hyperleukocytosis is significantly higher when ET therapy is used; however, this therapy has not yet been used in a pertussis patient in China.

In conclusion, hyperleukocytosis is strongly associated with fatal cases of pertussis in infants. Early recognition of pertussis-associated leukocytosis and treatment with appropriate leukoreduction therapy are critical for preventing mortality. This report highlights the importance of aggressive supportive care, as well as early implementation of ET, during the management of infants who are at a high risk of severe pertussis with hyperleukocytosis. Moreover, this report suggests that adults in China should be vaccinated against *B. pertussis* in order to prevent the infection of infants within their households.

## Acknowledgment

The authors thank the patient's father, who provided written permission for the publication of this case report.

## Author contributions

**Conceptualization:** Jikui Deng.

**Data curation:** Shufeng Tian, Hongmei Wang.

**Formal analysis:** Shufeng Tian.

**Methodology:** Shufeng Tian, Hongmei Wang.

**Project administration:** Jikui Deng.

**Visualization:** Jikui Deng, Hongmei Wang.

**Writing – original draft:** Shufeng Tian.

**Writing – review & editing:** Jikui Deng.
